# Detection and molecular characterization of *Clostridium perfringens, Paeniclostridium sordellii* and *Clostridium septicum* from lambs and goat kids with hemorrhagic abomasitis in Turkey

**DOI:** 10.1186/s12917-023-03569-5

**Published:** 2023-01-13

**Authors:** Hakan Kalender, Hasan Öngör, Necati Timurkaan, Burcu Karagülle, Burak Karabulut, Canan Akdeniz İncili, Hatip Enfal Başar, Elif Ekinci, Aydın Çevik, Eray Atıl, Burhan Çetinkaya

**Affiliations:** 1 Faculty of Veterinary Medicine, Department of Microbiology, Firat University, Elazig, Turkey; 2 Faculty of Veterinary Medicine, Department of Pathology, Firat University, Elazig, Turkey; 3 Faculty of Veterinary Medicine, Department of Pathology, Dicle University, Diyarbakir, Turkey; 4 Pendik Veterinary Control Institute, Istanbul, Turkey

**Keywords:** Hemorrhagic abomasitis, *Clostridium perfringens*, *Paeniclostridium sordellii*, *Clostridium septicum*, Lambs, Kids

## Abstract

**Background:**

The pathogenic Clostridia cause neurotoxic, histotoxic and enterotoxic infections in humans and animals. Several *Clostridium* species have been associated with abomasitis in ruminants. The present study aimed to investigate the frequency, and the presence of virulence genes, of *Clostridium perfringens, Paeniclostridium sordellii* and *Clostridium septicum* in lambs and goat kids with hemorrhagic abomasitis.

**Results:**

A total of 38 abomasum samples, collected from lambs and goat kids of 1 week to 1 month of age in different farms located in eastern Turkey between 2021 and 2022, were evaluated by histopathology, culture and PCR. At necropsy, the abomasum of the animals was excessively filled with caseinized content and gas, and the abomasum mucosa was hemorrhagic in varying degrees. In histopathological evaluation, acute necrotizing hemorrhagic inflammation was noted in abomasum samples. The examination of swab samples by culture and PCR revealed that *C. perfringens* type A was the most frequently detected species (86.84%) either alone or in combination with other *Clostridium* species. *P. sordellii*, *C. perfringens* type F and *C. septicum* were also harboured in the samples, albeit at low rates. Beta2 toxin gene (*cpb2*) was found in three of *C. perfringens* type A positive samples.

**Conclusion:**

It was suggested that vaccination of pregnant animals with toxoid vaccines would be beneficial in terms of protecting newborn animals against Clostridial infections. This study investigated the presence of clostridial toxin genes in abomasal samples for the first time in Turkey.

## Background

Clostridial diseases are common in livestock worldwide [1, 2]. The toxins produced by *Clostridium* species cause enteric, neurotoxic or histotoxic diseases in humans and animals [2]. Clostridial abomasitis and enteritis are responsible for extensive morbidity and high mortality rate in especially young ruminants [1]. Clostridial abomasitis is mostly caused by *Clostridium perfringens, Paeniclostridium sordellii* (previously known as *Clostridium sordelllii*) and *Clostridium septicum* species [3–11]. *C. perfringens* is classified into seven types (A-G) based on its capacity to encode six typing toxins, namely alpha, beta, epsilon, iota, enterotoxin and netB [12]. Each toxin type is associated with different enteric disaeases in animals. The beta2 toxin which is a non-typing toxin can be produced by different *C. perfringens* types [13]. It has been reported that beta2 toxin has a role in the pathogenesis of clostridial diseases [14, 15]. *C. perfringens* type A is associated with enterotoxemia in lambs (yellow lamb disease), and enteritis or enterotoxemia in cattle, pigs, horses, and goats, abomasitis in ruminants and hemorrhagic canine gastroenteritis [1, 2]. However, *C. perfringens* type A was mostly detected in cases of abomasitis in calves [5–8, 16]. *C. perfringens* type E has also been isolated from calves with abomasitis [17]. In addition to *Clostridium* species, *Sarcinia* spp., coccidiosis and copper deficieny have also been reported to be associated with abomasitis in animals [1, 18–21]. *P. sordellii* has been described as a cause of gas gangrene in animals [2, 22]. Recently, it has also been associated with abomasitis in lambs [1, 10, 11], enterocolitis in horses [23] and necrotic enteritis in chickens [24]. Lethal toxin (TcsL) and hemorrhagic toxin (TcsH) are mainly responsible for the virulence of *P. sordellii* [25, 26]. *C. septicum* causes abomasitis in sheep, known as braxy [2]. *C. septicum* has been isolated from ruminants with gas gangrene and abomasitis [2–4, 27]. It has been reported that alpha toxin is essential for the virulence of *C. septicum* [28].

Epidemiological investigations may help the development of vaccination programs against clostridial diseases and a better understanding of pathogenicity of *Clostridium* species. Although many studies have been conducted on the role of *Clostridium* species in the etiology of abomasitis in calves, there is a paucity of information in terms of etiology of abomasitis in lambs and goat kids. *P. sordellii* has been isolated from abomasum lesions of lambs in Turkey (11). The isolation of *C. perfringens* type A from lambs with enteric diseases has also been reported in Turkey and Iran (32, 35). However, to our knowledge, no studies are available concerning the presence of Clostridial toxin genes in abomasal samples in Turkey. This sudy was carried out to investigate the presence of *C. perfringens, Clostridium septicum* and *P. sordellii* and their toxin genes in abomasal samples of lambs and goat kids with hemorrhagic abomasitis in Turkey.

## Results

### Necropsy and histopathological findings

**Fig. 1:**
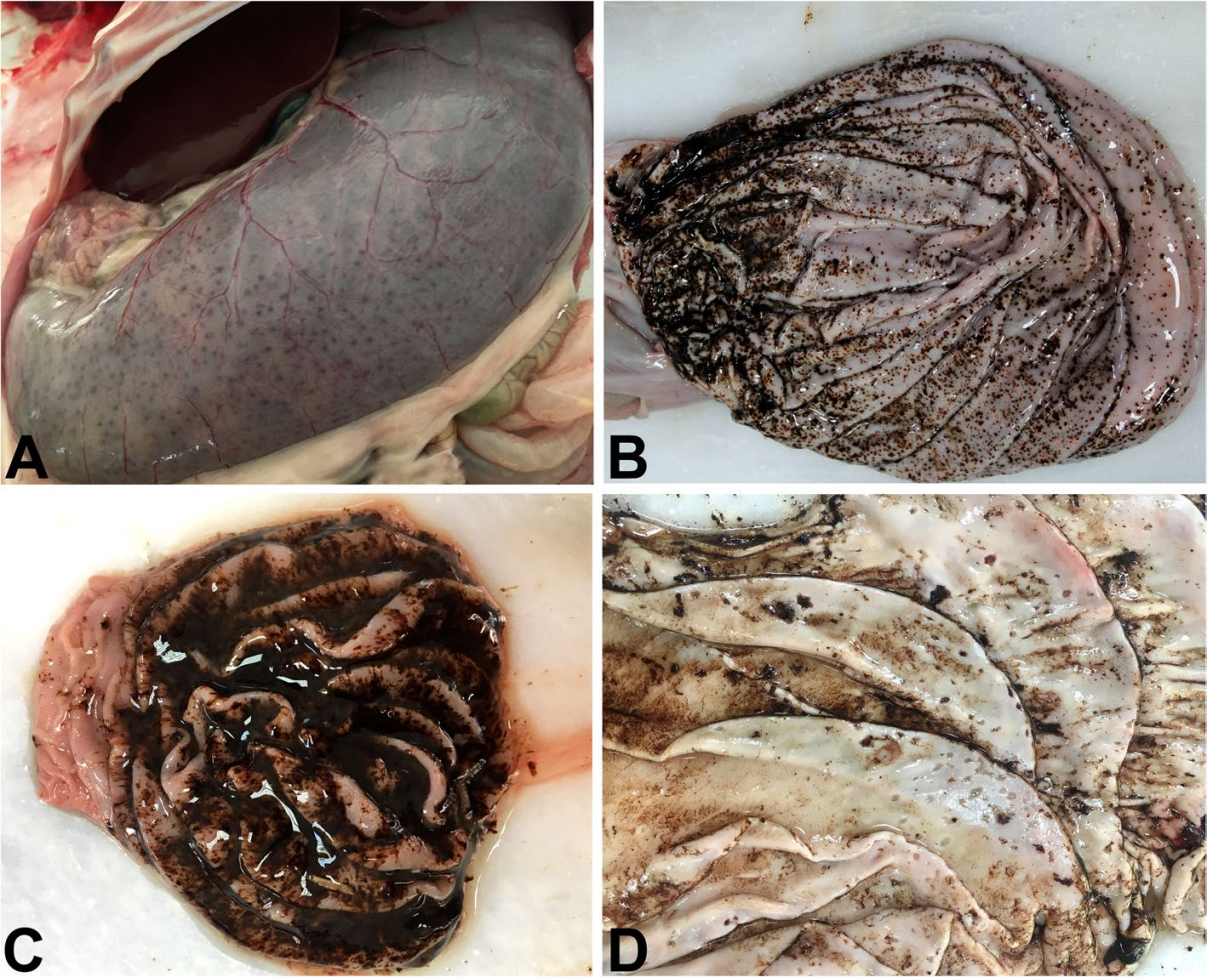
Macroscopic view of abomasums. **A**: Excessively distended abomasum with gas and caseinized contents, and multifocal hemorrhagic foci seen from serosa in a one-week old lamb. **B**: Multifocal severe petechyial hemorrhages on mucosal surface of the abomasum. **C**: The entire abomasum mucosa was covered with hemorrhagic content and abomasal wall were thickened duo to edema in a two-week old goat kid. **D**: Many erosions and ulcers were observed on the abomasal mucosa after the contents were removed in a ten-day old lamb

Necropsies revealed that gross findings were almost similar in all animals. The most striking finding at necropsy was that the abomasums of the animals were excessively dilated with caseinized content and gas, and multifocal black foci were seen in serosal surface (Fig. 1 A). When the abomasum mucosa was opened, it was observed that these black foci were petechial hemorrhages in varying degrees (Fig. 1B). The hemorrhages were quite severe in some animals and were observed to cover the entire abomasum mucosa like a layer, and the abomasal wall was thickened duo to edema (Fig. 1 C). Many erosions and ulcers were observed on the abomasal mucosa after the contents were removed (Fig. 1D). Ecchymotic hemorrhages were also observed in the epicardium and endocardium of the heart in addition to hemorrhages in the abomasum of five lambs and three kids.

**Fig. 2:**
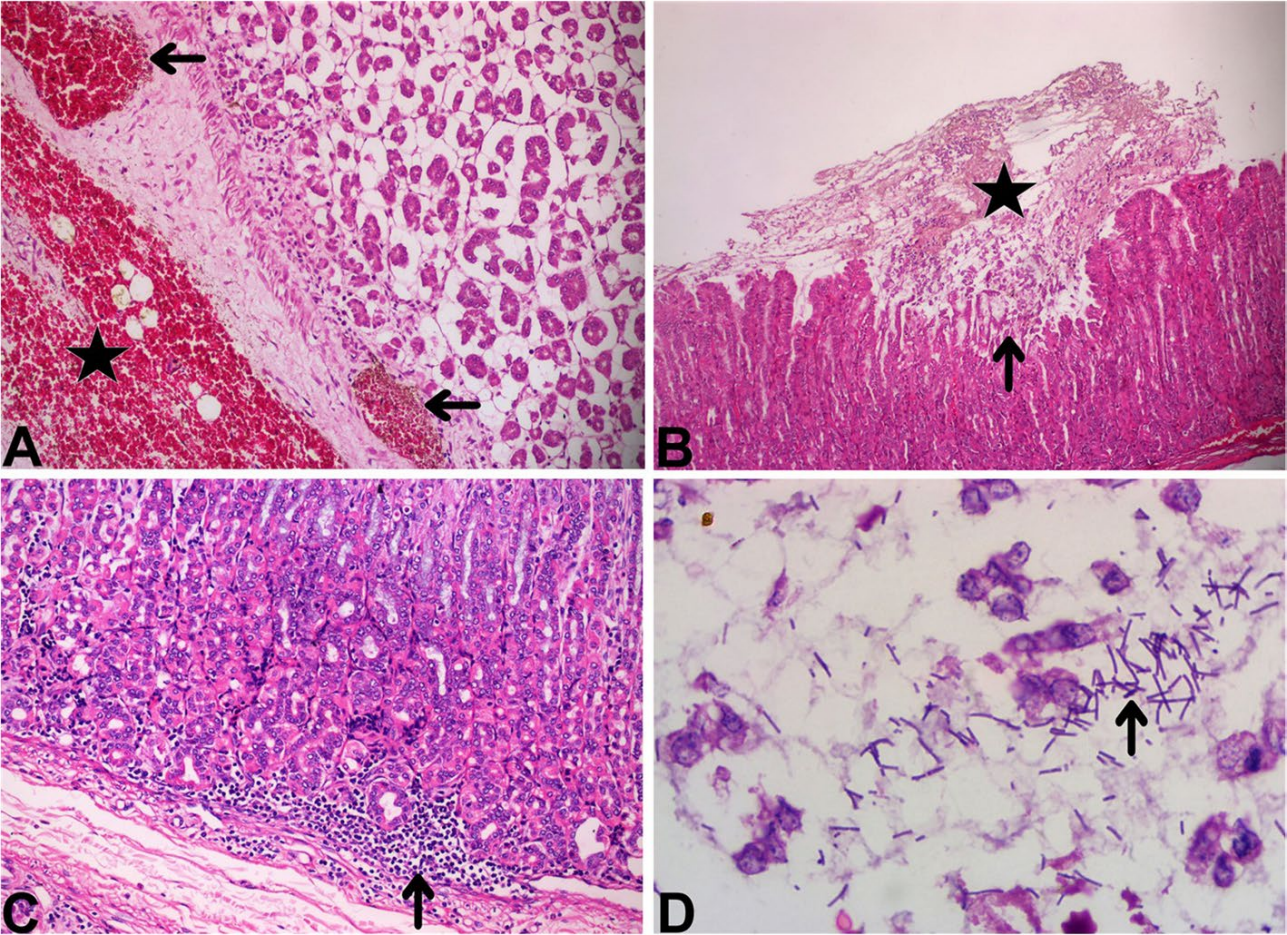
Microscopic views of abomasums. **A**: Hyperemie and/or congestion (arrows), and hemorrhagies (asterisk) were fonud in the propria mucosa and submucosa of a ten-day old lamb. H&E. 20 X Magnification. **B**: Mucosal erosion (arrow), and necrotic debris in the lumen of the abomasum (asterisk) in a 15-day old goat kids. H&E. 10 X Magnification. **C**: Mild numbers of neutrophils, lymphocytes and macrophages infiltrations in the propria mucosa of the abomasum (arrow) in a one-week old lamb. H&E. 20 X Magnification. **D**: Many large rod-shaped bacteria were found in the debris (arrow) of a two-week old lamb. H&E. 100 X Magnification

Acute necrotizing hemorrhagic inflammation of abomasum mucosa was observed at microscopic examination. Moderate or severe hyperemie, congestion, edema and hemorrhagies were found in the propria and submucosa (Fig. 2 A). There were erosions (Fig. 2B), some of which extending into submucosa ulcers characterized with epithelial degenerasion/necrosis and desquamation. Mild inflammatory infiltration including neutrophils, lymphocytes and macrophages was seen in the propria mucasa of the abomasums (Fig. 2 C). In addition, many large rod-shaped bacteria were detected in the debris and mucosal surface of abomasum in seven lambs and four kids (Fig. 2D). These bacteria were gram positive in the staining made by gram staining method.

## Molecular findings

Out of 38 samples, 33 (86.84%) were found positive for *C. perfringens* type A alone or in combination with other *Clostridium* species. *C. perfringens* type F (*cpa* and *cpe* gene positive) was detected in two (5.26%) samples. Other types of *C. perfringes* were not detected in any of the samples. Three samples were positive for *cpb2* in addition to *cpa*. *C. perfringens* type A and *P. sordellii* were detected in five (13.15%) samples. Two (5.26%) samples were positive for only *P. sordellii* and one (2.63%) sample for *C. perfringens* type A, *P. sordellii* and *C. septicum* (Table 1). The genes *tcsL and tcsH* were not detected in *P. sordellii* positive samples. One (2.63%) of the samples was found to be negative for either of these bacteria.

**Table 1:** The distribution of *Clostridium* species detected in abomasal swab samples

Species	Sample No	No. (%) of positive sample				
		*C. perfringens* type A	*C. perfringens* type F	*P. sordellii*	*P. sordellii* + *C. perfringens* type A	*C. septicum* + *P.sordellii* + *C. perfringens* type A
Lamb	21	15 (71.42)	1 (4.76)	1 (4.76)	3 (14.28)	1 (4.76)
Goat kid	17^*^	12 (70.58)	1 (5.88)	1 (5.88)	2 (11.76)	-
**Total**	**38**	**27 (71.05)**	**2 (5.26)**	**2 (5.26)**	**5 (13.16)**	**1 (2.63)**

^*^: Clostridium spp. was not isolated in one sample

## Discussion

Clostridial abomasitis is characterized by necrosis of abomasal mucosa caused by exotoxins produced by several species of *Clostridium* genus in the gastrointestinal tract of animals [1]. Overgrowth of *Clostridium* species within the gastrointestinal tract and subsequent exotoxin release may lead to sudden death in animals under predisposing conditions [2, 29]. Factors such as overfeeding, contamination of pooled colostrum, poor hygiene in farms, changes in diet, mineral deficiencies and formation of trichophytobezoars predispose to abomasal bloat and abomasitis in young ruminants [2]. Although *C. septicum* is traditionally associated with abomasitis in ruminants, the role of other clostridial agents is not well understood. The present study aimed to investigate the frequency, and the presence of virulence genes, of *C. perfringens, P. sordellii* and *C. septicum* in lambs and goat kids with hemorrhagic abomasitis. The vast majority of the 38 abomasal samples were determined to harbour *C. perfringens* type A. In addition, *P. sordellii*, *C. perfringens* type F and *C. septicum* were detected in the samples, albeit at low rates. Meanwhile, a few samples contained more than one *Clostridium* species. These findings put forward that *C. perfringens* type A has an important role in the etiology of hemorrhagic abomasitis in small ruminants. Recently, *C. perfringens* type A was increasingly isolated from calves with abomasitis [6–8]. In the USA, *C. perfringens* was reported to be present in approximately 67% of calves died of emphysematous abomasitis and abomasal bloat [16]. On the other hand, the number of studies reporting the presence of *C. perfringens* in abomasitis cases of lambs and goat kids was rather limited. In accordance with our results, *C. perfringens* type A was detected in 84% of the cases with clostridial enterotoxaemia in lambs and goat kids in Italy [15]. In another study carried out in Pakistan, the majority of the *C. perfringens* isolates from sheep and goats (healthy and diseased) were characterized as type A (82%) [30]. Likewise, 81% of *C. perfringens* isolates obtained from domestic livestock were reported to belong to type A in Saudi Arabia [31]. In Turkey, 77% of *C. perfringens* isolates from lambs suspected of enterotoxemia were genotyped as type A [32]. *C. perfringens* types B, C and D toxoid vaccines produced in Turkey are used against *C. perfringens* infections in animals. However, the results of the current study suggest that *C. perfringens* type A toxoid vaccine should also be considered in the vaccination programs of sheep and goats.

The major toxin of *C. perfringens* type A is alpha toxin which is a zinc metallophospholipase. High concentrations of this toxin can damage plasma membrans of host cells [2]. It was reported that alpha toxin might contribute to pathogenesis of bovine necro-hemorrhagic enteritis [33]. However, the role of *C. perfringens* type A in the pathogenesis of enteric disease of animals is still conroversial, as it can be found in the intestine of clinically healthy animals [34]. In recent years, beta2 toxin has frequently been detected in *C. perfringens* isolates from animals and humans [14, 15, 35]. Also, a significant relationship has been reported between *cpb2*-positive *C. perfringens* isolates and diarrhoea in pigs [36]. This toxin has been detected in horses with intestinal disorders, as well [37]. In the present study, although only three of *C. perfringens* type A positive samples were found to harbour *cpb2*, it can be suggested that beta2 toxin might act in sinergy with alpha toxin in the pathogenicity of *C. perfringens* type A [1, 14, 33]. However, it should be underlined that some studies failed to detect *cpb2* in *C. perfringens* type A isolates from either bovine clostridial abomasitis (BCA) or jejunal hemorhage syndrome (JHS) [7, 31].

According to a recently introduced toxin-based typing system, *C. perfringens* strains containing *cpa* and *cpe* genes were classified as type F, while those containing *cpa* and *netB* genes were as type G [12]. *C. perfringens* type F is associated with food poisoning in humans. However, a few enterotoxigenic infections caused by type F strains have been reported in animals [38, 39]. Although the presence of *cpe* has been reported in a goat with necrotizing enterocolitis [40], the role of enteroxin producing *C. perfringens* strains in animal diseases is not enlightened [39]. Our results suggested that enterotoxin does not have an important role in hemorrhagic abomasitis cases, but further studies are needed to clarify the pathogenicity of enterotoxin in animal diseases. *C. perfringens* type G causes necrotic enteritis in poultry [2]. The presence of this type has also been reported in a cow [41]. However, in accordance with many previous studies [7, 14, 42, 43], none of the samples were positive for *C. perfringens* type G in the present study.

Although *P. sordellii* is known to cause gas gangrene in animals [2], it has also been isolated from lambs with abomasitis [10, 11, 44]. In the current study, *P. sordellii* was detected as the second most common bacterial species after *C. perfringens* type A. Lethal and hemorrhagic toxins (TcsL and TcsH) are considered to be the main virulence factors of *P. sordellii*. These toxins are members of the Large Clostridial Cytotoxin (LCC) family [45, 46]. Although at low rates, the presence of LCC genes in *P.sordellii* strains isolated from clinical cases including horses with enterocolitis has been demonstrated in the UK, USA and Australia [23, 46]. However, this study failed to show these genes in *P. sordellii* isolates. Similarly, Zerrouki et al. [47] reported that *P. sordellii* isolates originated from a hospital environment in Algeria did not harbour either TcsL or TcsH toxins. The absence of these genes in our isolates was not surprising due to the facts that the number of isolates was small (*n* = 8) and that the majority of *P.sordellii* strains do not encode LCC genes [46]. It is therefore possible that additional virulence factors can contribute to the pathogenicity of *P. sordellii.* However, it is known that nontoxigenic strains of *P.sordelli* can cause the disease, albeit at low severity [48, 49].

*Clostridium septicum* is considered as the etiological agent of braxy in sheep [2]. It has been determined in lambs and calves with suppurative abomasitis [4, 9]. Acute haemorrhagic abomasitis due to *C. septicum* has also been reported in experimental sheep [3]. However, the results of this study suggested that *C. septicum* was not the primary agent of abomasitis in lambs and goat kids in Turkey. It is known that small ruminants are mostly vaccinated against braxy in Turkey. Therefore, the detection of this agent in only one sample in the present study may be attributed to passive immunity in newborns. Similar results have also been reported elsewhere. For instance, *C. septicum* was not detected from lambs aged between 2 and 5 weeks with abomasal haemorrhage and ulcers in Norway [10].

Other bacterial agents such as *Clostridium fallax* have rarely been reported in abomasitis cases of lambs [10, 20]. Recently, *Sarcinia* genus bacteria have also isolated from lambs and calves with abomasitis [10, 19, 20]. In the present study, the samples were not analyzed for either *C. fallax* or *Sarcinia* spp. However, the organisms consistent with *Sarcinia* spp. were not observed on the surface or within the abomasum wall in the histopathological examination.

## Conclusion

The results of this study showed that *C. perfringens* type A was the most common species followed by *P. sordellii* in lambs and goat kids with hemorrhagic abomasitis in eastern Turkey. Interestingly, *C. septicum* was detected at the lowest rate in the abomasal samples, probably due to widespread vaccination of mothers. Also, the present study provided data concerning clostridial toxin genes in abomasum samples for the first time in Turkey. Further comprehensive studies are needed to have a better understanding of the pathogenic mechanisms of *Clostridium* species in abomasitis of small ruminants.

### Methods

#### Sampling

The study material consisted of 21 lambs and 17 goat kids aged between 1 week and 1 month belonging to 30 different farms located in Eastern Turkey, which were submitted to the Pathology Department of Firat University between 2021 and 2022. Sudden onset of weakness, inability to suck the mother, reluctance to move or rise, swelling in the abdomen and death within 2–3 h to 1 day were recorded as general complaints in animals that were born healthy and received sufficient amount of colostrum. The mortality rate of the offsprings in the abovementioned age groups was informed to be around 15–20% in the flocks.

### Necropsy and histopathological examination

Systematic necropsies were performed, tissue samples were taken from all animals, and fixed in 10% buffered neutral formalin solution. After routine procedures, the prepared paraffin blocks were cut into 3 μm thick, stained with haematoxylin and eosin (H&E), Brown and Brenn method for gram staining, and were evaluated by light microscopy.

### Bacteriological culture

The swab samples were cultured onto 5% blood agar and incubated in an anaerobic jar for 48 h at 37 °C. Following Gram staining, suspected colonies were subcultured into cooked meat medium and incubated for 48 h at 37 °C in anaerobic conditions. Anaerobic media were supplied with anaerobic gas kits (Anaerocult A, Merck).

### DNA extraction

DNA extraction was carried out from the bacterial culture in the cooked meat medium. For this, 300 µL of bacterial cultures were transferred to Eppendorf tubes. Each sample was treated with 300 µl TNES buffer (20mM Tris, 150 mM NaCl, 10 mM EDTA, 0.2% SDS) and 6 µl proteinase K (20 mg/ml), and then inactivated at 56 °C for 2 h. After the mixture was boiled for 10 min., 600 µL phenol-chloroform-isoamyl alcohol was added. The mixture was shaken vigorously for 5 min and centrifuged at 11600x g for 10 min. The upper phase was transferred to another Eppendorf tube and 3 M sodium acetate (0.1 volume) and ethanol (2.5 volume) were added. The mixture was vortexed and kept at -20 °C overnight. The mixture was then centrifuged at 11600x g for 10 min, and supernatant was removed. The pellet was washed with 70% ethanol and centrifuged at 11.600 g for 5 min. Finally, the pellet was dried for 45 min and suspended in 50 µL distilled water.

## Polymerase chain reaction (PCR)

*C. perfringens* NCTC 8239 (*cpa*^*+*^ and *cpe*^*+*^), *C. perfringens* NCTC 13,110 (*cpa*^*+*^, *cpb*^*+*^ *cpb2* and *etx*^*+*^), *C. perfringens* CCUG 44,727 (*cpa*^*+*^ and *itx*^*+*^), *C. septicum* genomic DNA and *P. sordellii* genomic DNA were used as positive controls. A multiplex PCR was performed for the detection of alpha (*cpa*), beta (*cpb*), epsilon (*etx*), iota (*itx*), enterotoxin (*cpe)* and beta2 (*cpb2*) toxin genes of *C. perfringens* in a thermal cycler (Techne, Staffordshire, UK), in a total reaction volume of 25 µL containing 12.5 µL 2X AmpMasterTaq master mix (AmpMasterTMGeneAll Biotechnology, Cambiol, Cambridge, Cat No: 541 − 010), 2.5 µL of DNA, and 1 µL of each specific primer (10 µM). The amplified products were electrophoresed in a 1.5% agarose gel containing 10 µL ethidium bromide solution. Amplified products were visualized and photographed under UV light. PCR products with the molecular sizes of approximately 402 bp, 196 bp, 567 bp, 655 bp, 293 and 506 bp were considered positive for *cpa, cpb, cpb2, etx, itx* and *cpe* genes, respectively. A single PCR was employed for the detection of the *C. perfringens* necrotic enteritis B-like toxin (*netB*) gene. A primer pair specific for the flagellin gene (*fliC)* was used for the identification of *C. septicum* by a single PCR. The *C. septicum* positive sample was also screened for the presence of alpha toxin (Hemolysin) gene. For the identification of *P. sordellii*, the samples were tested with PCR as previously described [50]. The primers of JRP4589 and JRP4590 were used to amplify a fragment of approximately 470 bp, representing an internal region of *sdlO* gene. PCR was also performed to determine the presence of the lethal toxin (*tcsl*) and hemorrhagic toxin (*tcsh*) genes of *P. sordellii* as previously described [29]. The primers used in this study were listed in Table 2.

**Table 2:** DNA sequences of primers used in this study

Target gene	Sequence (5’-3’)	Amplicon size (bp)	References
*C. perfringens*			
* cpa*	GTTGTAAGCGCAGGACATGTTAAG CATGTAGTCATCTGTTCCAGCATC	402	[51]
* cpb*	GCGAATATGCTGAATCATCTA GCAGGAACATTAGTATATCTTC	196	[52]
* etx*	CGGGTGATATCCATCTATTC CCACTTACTTGTCCTACTAAC	655	[52]
* itx*	AAACGCATTAAAGCTCACACC GTGCATAACCTGGAATGGCT	293	[52]
* cpe*	GGGGAACCCTCAGTAGTTTCA ACCAGCTGGATTTGAGTTTAATG	506	[53]
* cpb2*	AGATTTTAAATATGATCCTAACC CAATACCCTTCACCAAATACTC	567	[54]
* netB*	GCTGGTGCTGGAATAAATGC TCGCCATTGAGTAGTTTCCC	384	[55]
*P. sordellii*			
* sdlO*	TTACAGTTCAAAACCCAACCTATGG TGCAGCTTGTACATCTTTGCTCTTA	470	[24]
* tcsl*	AGAATGTGAGATAAATGTTGCTTCA ATCCTAAATCCATTTTCAGTCTTGG	228	[23]
* tcsh*	ATTGTGGCACGAGCTTCTGG TCCAGCTATAGAATTAGGTGGCA	153	[23]
*C. septicum*			
Alpha toxin	AATTCAGTGTGCGGCAGTAG CCTGCCCCAACTTCTCTTTT	270	[27]
* fliC*	AGAATAAACAGAAGCTGGAGATG TTTATTGAATTGTGTTTGTGAAG	294	[27]

## Data Availability

The data supporting our findings are contained within the manuscript.

## Abbreviations

HE: Hematoxylin-eosin
PCR: Polymerase chain reaction
